# Baohe pill decoction treats diarrhea induced by high-fat and high-protein diet by regulating lactase-producing bacteria in intestinal mucosa

**DOI:** 10.3389/fmicb.2023.1157475

**Published:** 2023-05-09

**Authors:** Kang Zhou, Xin Yi, Zhoujin Tan, Maijiao Peng, Nenqun Xiao

**Affiliations:** ^1^College of Pharmacy, Hunan University of Chinese Medicine, Changsha, China; ^2^College of Chinese Medicine, Hunan University of Chinese Medicine, Changsha, China

**Keywords:** Baohe pill decoction, lactase-producing bacteria, intestinal musoca, high-fat and high-protein diet, diarrhea

## Abstract

**Introduction:**

This study aimed to investigate the effects of Baohe pill decoction (BPD) on microbial, lactase activity, and lactase-producing bacteria in the intestinal mucosa of mice with diarrhea induced by high-fat and high-protein diet (HFHPD).

**Methods:**

Thirty male Kunming (KM) mice were randomly divided into normal (NM), model (MD), and BPD groups. Diarrhea models were manufactured using HFHPD combined with a gavage of vegetable oil. At the end of modeling, the BPD group was given BPD (6.63 g·kg^−1^d^−1^) intervention twice daily for 3 d. The NM and MD groups were given equal amounts of sterile water. Subsequently, the intestinal mucosa of the mice was collected, one portion was used for microbial and lactase activity measurement, and the other portion was used for its lactase-producing bacterial characteristics by high-throughput sequencing technology.

**Results:**

Our results showed that microbial and lactase activity of intestinal mucosa decreased significantly following diarrhea in mice (*P*_microbial_ < 0.05, *P*_lactase_ < 0.001). After BPD intervention, microbial and lactase activity increased significantly (*P* < 0.01). The number of operational taxonomic units (OTUs), richness, and diversity index of lactase-producing bacteria increased in the BPD group compared to the MD group (*P* > 0.05), and the community structure were significant differences (*P* < 0.01). Compared to other groups, *Saccharopolyspora*, *Rhizobium*, *Cedecea*, and *Escherichia* were enriched in the BPD group. Notably, the relative abundance of the dominant lactase-producing genus *Bifidobacterium* decreased after BPD intervention.

**Discussion:**

The mechanism of BPD in relieving diarrhea induced by HFHPD is closely related to the promotion of lactase activity in the intestinal mucosa, which may be achieved by regulating the structure of lactase-producing bacteria.

## 1. Introduction

The intestinal mucosa is the first point of contact between the ingested diet and the intestine and also serves as an immune barrier separating the host’s internal and external environments. It aids in food digestion, nutrient absorption, and immune sensing, but also restricts the entry and exit of harmful antigens and microorganisms (Vancamelbeke and Vermeire, 2017). Meanwhile, commensal microbiota inhabiting the mucus layer also promotes immune cell development, enhances intestinal epithelial cell (IEC) tight junctions, which shape the intestinal immune system together with the intestinal mucosa, and participate in the host immune response (Zmora et al., 2019; Gasaly et al., 2021). Diet is the primary factor shaping the structure and function of the intestinal microbial community (Shalapour and Karin, 2020). By interacting directly or indirectly with the intestinal microbiota to produce new bioinformatic factors, different dietary patterns can cause different degrees of beneficial or detrimental effects on the host’s physiological and pathological conditions. Among others, a high-fat or high-protein diet can increase the permeability of the intestinal mucosa, leading to impaired intestinal mucosal barrier function (Mittendorfer et al., 2020; Ren et al., 2022). In previous mouse experiments (Shao et al., 2022; Zhu et al., 2022), we found that HFHPD causes a significant decrease in the activity of several intestinal enzymes, including lactase, as well as structural changes in lactase-producing bacteria in the intestinal mucosa and a decrease in the abundance of beneficial bacteria, which, in turn, leads to diarrhea.

Lactase is also called β-galactosidase. The villi of intestinal mucosal epithelial cells and some lactase-producing microorganisms are the endogenous sources of lactase in the body (Facioni et al., 2020). If its activity is reduced or absent, excess lactose will be fermented by intestinal microorganisms, causing adverse reactions such as diarrhea and flatulence, which is clinically called lactose intolerance (Facioni et al., 2020). Lactase is encoded by functional genes, and its activity is genetically controlled, which means that the stability of the intestinal microbiota and mucosal barrier has an important influence on lactase activity. In addition, the self-regulatory ability of the intestinal mucosa can also promote the homeostatic balance of the intestinal microbiota, which, in turn, provides a stable expression environment for microbial enzyme proteins. In fact, it has also been proved that almost all conditions that may lead to intestinal injury, including antibiotic-induced diarrhea (AAD), acute gastroenteritis, and Giardia growth, will affect lactase activity, thus inducing lactose malabsorption (Wanes et al., 2019). Fortunately, lactase or lactase-producing bacteria supplementation can effectively alleviate diarrhea caused by low lactase activity (Ibrahim et al., 2021). Moreover, a variety of Traditional Chinese medicine (TCM) compounds have been found to treat diarrhea by affecting the activity of intestinal lactase, such as Qiwei Bai Zhu San (Hui et al., 2018), Tongxie Yaofang (Wu et al., 2020), and compound *Radix pulsatillae* (Zhang et al., 2010). The preliminary studies showed that it was closely related to the regulation of lactase-producing bacteria. For example, AAD changed or damaged the community structure, diversity, and relative abundance of lactase-producing bacteria in the intestinal tract of mice to different degrees, and Qiwei Baizhu Powder could alleviate AAD by promoting the growth of key lactase-producing bacteria (Long et al., 2017a, 2018a,b; He et al., 2018).

Baohe pill is a TCM compound applied to food accumulation syndrome caused by improper or excessive diet, which is composed of seven herbs, such as *Crataegus pinnatifida* Bge., *Pineilia ternata* (Thunb.) Breit., and *Poria cocos* (Schw.) Wolf (He et al., 2020). The main effective components include hesperidin, forsythin, oleanolic acid, and ursolic acid (He et al., 2020). Previous studies have shown that BPD plays a good role in the repair of intestinal mucosal injury, the recovery of intestinal microorganisms and the regulation of intestinal enzymes (including lactase; You et al., 2021; Guo et al., 2022; Huang et al., 2022). For example, BPD can treat diarrhea caused by HFHPD by increasing the abundance of the lactase-producing bacterium *Lactobacillus* spp., while reducing the abundance of the opportunistic pathogen *Ralstonia* (Huang et al., 2022). However, there are few studies on the efficacy of disease-related functional enzyme genes in TCM compound prescriptions. In addition, due to regional microecological differences, compared with the content microbiota, the intestinal mucosal microbiota was more stable, not easily affected by external factors such as food, and was more closely related to the intestinal mucosa. Hence, the results of its flora changes were more representative (Zhou et al., 2022a). In early studies (Zhou et al., 2022b), we studied the relationship between BPD and lactase-producing bacteria in the intestinal content, but a potential association with lactase-producing bacteria in the intestinal mucosa has yet to be identified. In this study, we explored the effect of BPD on the lactase-producing bacteria in the intestinal mucosa of mice with diarrhea induced by HFHPD, and detected the lactase and microbial activity in the intestinal mucosa, aiming to reveal the curative mechanism of BPD in the treatment of diarrhea caused by unhealthy diet pattern from the level of disease-related microbial enzyme gene, which will provide new ideas for the research on the curative mechanism of BPD and new targets of drugs.

## 2. Materials and methods

### 2.1. Animals and diet

Thirty 3-week-old KM mice weighing 20 ± 2 g were purchased from Hunan Slaccas Jingda Laboratory Animal Company. To exclude sex differences, all the above mice were male (Wu et al., 2022). The mice were raised throughout in the barrier facility (relative humanity: 50%–70%, temperature: 23°C–25°C) at the laboratory animal center of the Hunan University of Chinese Medicine [SYXK (Hunan) 2019-0009]. The general feed was provided by the laboratory animal center of the Hunan University of Chinese Medicine (20% protein, 4% fat). The high-fat and high-protein feed was prepared by oneself, which was prepared by mixing milk (Nestle, 30% protein, 20% fat), soybean powder (Huiyi, 33% protein, 18% fat), flour (Huiyi, 13% protein, 2% fat), and minced meat (Anhui Lizheng, 30% protein, 25% fat) in a ratio of 1:2:2:1. Vegetable oil (Golden Dragonfish, 59% soybean oil, 21% rapeseed oil, 10% sunflower seed, 3% peanut oil, 3% rice oil, 3% corn oil, 0.6% sesame oil, and 0.4% linseed oil). All experiments and procedures involving animals were performed according to the protocols approved by the Institutional Animal Care and Use Committee of the Hunan University of Chinese Medicine.

### 2.2. Medicine

Composition of Baohe pill: *Crataegus pinnatifida* Bge. (18 g, Hebei), medicated leaven (6 g, Sichuan), *Pineilia ternata* (Thunb.) Breit. (9 g, Sichuan), *Poria cocos* (Schw.) Wolf (9 g, Hunan), *Citrus reticulata* Blanco (3 g, Zhejiang), *Raphanus sativus* L. (3 g, Anhui), and *Forsythia suspensa* (Thunb.) Vahl (3 g, Shanxi). After the seven materials were weighed according to the proportion of the whole prescription, 300 mL of water was added, and the mixture was boiled for 30 min, followed by filtration, to collect the first filtrate. After 200 mL of water was added to the filter residue, the mixture was boiled for 30 min, filtered, and the filtrates were combined and concentrated into the BPD with a crude drug concentration of 0.28 g/mL, which was stored in a −4°C refrigerator for subsequent use (Guo et al., 2022).

### 2.3. Reagents

Acetone, Hunan Huihong Reagent Co., Ltd.; Fluorescein diacetate (FDA) and Ortho-nitrophenyl beta-D-galactopyranoside (ONPG) were purchased from Shanghai Yuanye Biotechnology Co., Ltd. Proteinase K, TE buffer, lysozyme, chloroform:isoamyl alcohol (24:1), Tris-saturated phenol:chloroform:isoamyl alcohol (25:24:1), and acetone were purchased from Beijing Dingguo Biotechnology Co., Ltd. 0.1 mol/L PBS buffer, 10% SDS, 5 mol/L NaCl, CTAB/NaCl, 3 mol/L sodium acetate, and 70% anhydrous ethanol, etc. were configured by the laboratory. FDA stock solution configuration: add 2 mg of FDA to 1 mL of acetone solution, mix well to obtain 2 mg/mL of FDA stock solution, and store in the dark at −20°C. FDA reaction solution configuration: the FDA stock solution was added to PBS buffer (pH = 7.6) for a final FDA concentration of 10 μg/mL (Li et al., 2023).

### 2.4. Grouping, modeling, and administration of experimental animals

**Figure 1:**
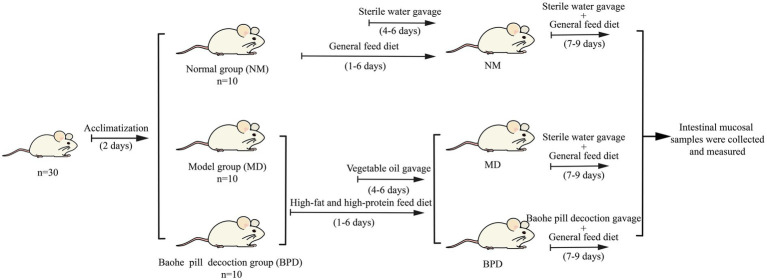
Animal experimental design.

The 30 mice were randomly divided into the normal group (NM), model (MD), and BPD group, with 10 mice in each group and 5 mice in one cage, with the numbers of NM1-10, MD1-10, and BPD1-10, respectively. As shown in Figure 1, after adaptive feeding for 2 days, both MD and BPD groups were given an HFHPD, and on day 4, vegetable oil was gavage at 0.4 mL twice/d for 3 days. NM group was given a general diet and gavage with equal sterile water in place of vegetable oil. After 6 days of HFHPD intervention, and the BPD group was treated with BPD according to the conversion of mouse body surface area, i.e., 6.63 g·kg^−1^d^−1^ for 3 days. NM and MD were gavage with equal amounts of sterile water (Shao et al., 2022).

### 2.5. Sampling

After rapid euthanasia by cervical dislocation in three groups of mice, the intestinal mucosa was removed from the jejunum to ileum section of the mice, dissected and rinsed in physiological saline, placed on sterile filter paper to absorb the water, scraped on weighing paper, and stored frozen at −80°C (Zhang et al., 2020). Five mouse intestinal mucosa samples were randomly selected from each group and mixed for microbial and lactase activity assay. The remaining five were used for the sequencing of lactase-producing bacteria.

### 2.6. Microbial and lactase activity measurement

The intestinal mucosa was weighed, placed in sterile centrifuge tubes with glass beads, added with the appropriate amount of saline, shaken in a shaker for 30 min (120 r/min), centrifuged for 10 min (3,000 r/min), and the supernatant was used for microbial activity and lactase activity assay. The supernatant was used for the determination of microbial activity and lactase activity. The microbial activity was measured by the FDA method. Three sterile tubes were used for three parallel replicates, and 2 mL of FDA reaction solution and 50 μL of the sample supernatant were added, mixed evenly, and shaken at 24°C. Shake for 90 min, add 2 mL of acetone, shake well, and measure the absorbance of the sample at OD_490_. Another sterile tube was taken as a blank control, 2 mL of the reaction solution and 2 mL of acetone were added, then 50 μL of the sample supernatant was added, and the absorbance was measured under the same conditions after a shake for 90 min. The lactase activity was measured by the ONPG method. The amount of enzyme generated by hydrolysis ONPG at 37°C per minute from 1 g of intestinal mucosa to 1 mmol of o-nitrophenol was used as one unit of enzyme activity.

### 2.7. DNA extraction and PCR amplification

Total bacterial DNA was obtained by acetone washing, proteinase K denaturation, SDS lysis, and phenol/chloroform extraction, referring to the literature (Shao et al., 2020) for the specific steps. Subsequently, amplification was performed using the lactonase-producing bacterial primers we reported previously (Long et al., 2017b), i.e., forward primer: 5′-TRRGCAACGAATACGGSTG-3′ and reverse primer: 5′-ACCATGAARTTCSGTGGTSARCGG-3′. The PCR amplification system is shown in Table 1. PCR amplification conditions: initial denaturation at 98°C for 30 s and then entering the amplification cycle. Denaturation at 98°C for 15 s, annealing at 46°C for 30 s, and extension at 72°C for 30 s. After 32 cycles, it extended for 5 min at 72°C and stored at 4°C. The sequencing was performed by Shanghai Paisennuo Biological Technology Co., Ltd.

**Table 1:** PCR amplification system.

PCR amplification system	Volume (μL)
Q5 high-fidelity DNA polymerase	0.25
5 × reaction buffer	5
5 × high GC buffer	5
dNTP (10 mM)	0.5
Template DNA	1
Forward primer (10 uM)	1
Reverse primer (10 uM)	1
Ultrapure water	11.25

### 2.8. Bioinformatic analysis

Vsearch (v 2.13.4) and cutadapt (v 2.3) software were used to splice, de-duplicate, filter the resulting sequences, and divide the OTUs with a 97% similarity threshold (Blaxter et al., 2005). The delineated OTU representative sequences were compared with the NCBI database on Qiime2 (v 2019.4) software to obtain OTU species annotation information. Diversity indices such as Chao1, Observed species, Shannon, Simpson, Pielous evenness, and Goods coverage indices were calculated using Qiime2 and visualized using R (v 4.2.2). Goods coverage indices were used to assess the proportion of species obtained from sequencing to the total number of species in the sample, Chao1, Observed species indices to assess community richness, and Shannon, Simpson, and Pielous evenness indices to assess community evenness. Principal coordinate analysis (PCoA) was used to assess community structure, and we used Adonis test to express the difference between communities, with *p* < 0.05 considered as the significant difference (Caporaso et al., 2010).

### 2.9. Statistical analysis

Experimental results data were expressed as mean ± standard deviation (x ± s). Data were analyzed with IBM SPSS (v 25.0) software, and when the data conformed to the normal distribution and variance uniformity, one-way ANOVA test was used to compare the differences among multiple groups. The LSD method was used for pairwise comparison between groups. When the data did not conform to the normal distribution or variance was inconsistent, the Kruskal–Wallis test in non-parametric test and Bonferroni correction were used. *p* < 0.05 was considered a significant difference.

## 3. Results

### 3.1. General behavioral observations in mice

The mice in the NM group were in good mental condition and had normal feces. Mice in the MD and BPD groups had decreased glossy fur, depressed mental condition, showed diarrhea symptoms, unshaped soft sludge-like feces, filthy perianal area and visible fecal adhesion. After the intervention of BPD, the mice had restored shiny fur, increased voluntary activity, and their feces changed from soft and rotten to normal. The naturally recovered mice in the MD group also converged to the state of the mice in the NM group, but individual mice were still in poor mental and had unformed stools.

### 3.2. Effect of BPD on microbial and lactase activity in the intestinal mucosa of mice with diarrhea induced by HFHPD

**Figure 2:**
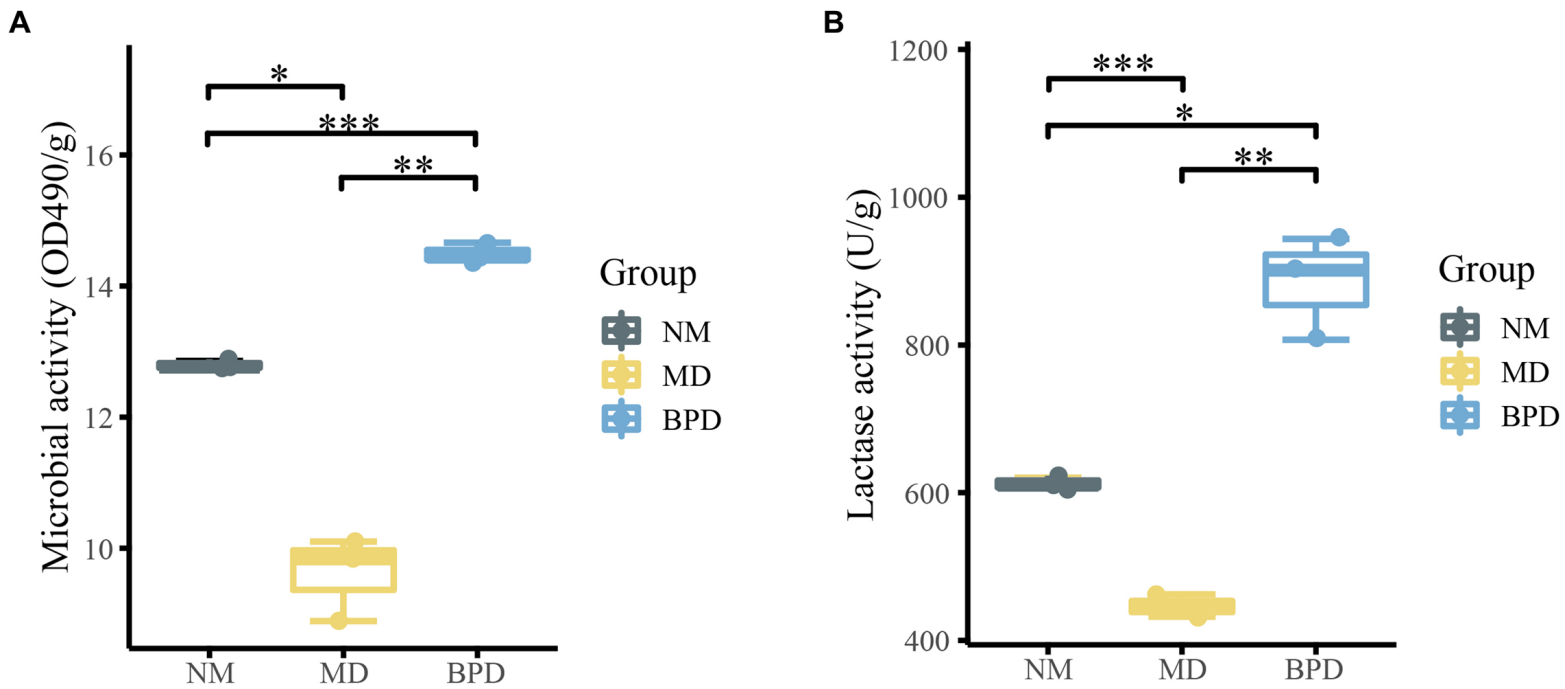
Microbial and lactase activities of intestinal mucosa in mice. **(A)** microbial activity; **(B)** lactase activity. NM, normal group; MD, model group; BPD, Baohe pill decoction group. ^*^p < 0.05, ^**^p < 0.01, ^***^p < 0.001.

FDA can be hydrolyzed by non-specific enzymes expressed by bacteria and fungi, while its degree of hydrolysis is proportional to microbial activity, so the total microbial activity of the intestinal mucosa can be assessed by detecting the degree of hydrolysis of FDA (Swisher and Carroll, 1980). From Figures 2A,B, the microbial activity was consistent with the change in lactase activity. Microbial and lactase activity was significantly lower in the MD group compared to the NM group (*P*_microbiota_ < 0.05, *P*_lactase_ < 0.001). The microbial activity and lactase activity in the BPD group were significantly increased after the intervention of BPD (*P*_microbiota_ < 0.01, *P*_lactase_ < 0.01). Meanwhile, microbial and lactase activity was significantly higher in the BPD group than in the NM group (*P*_microbiota_ < 0.001, *P*_lactase_ < 0.05). This indicates that HFHPD modeling reduced the microbial and lactase activity of mouse intestinal mucosa, while BPD had a significant restoring and promoting effect on their microbial and lactase activity.

### 3.3. Sequencing quality and number of OTUs for each group

**Figure 3:**
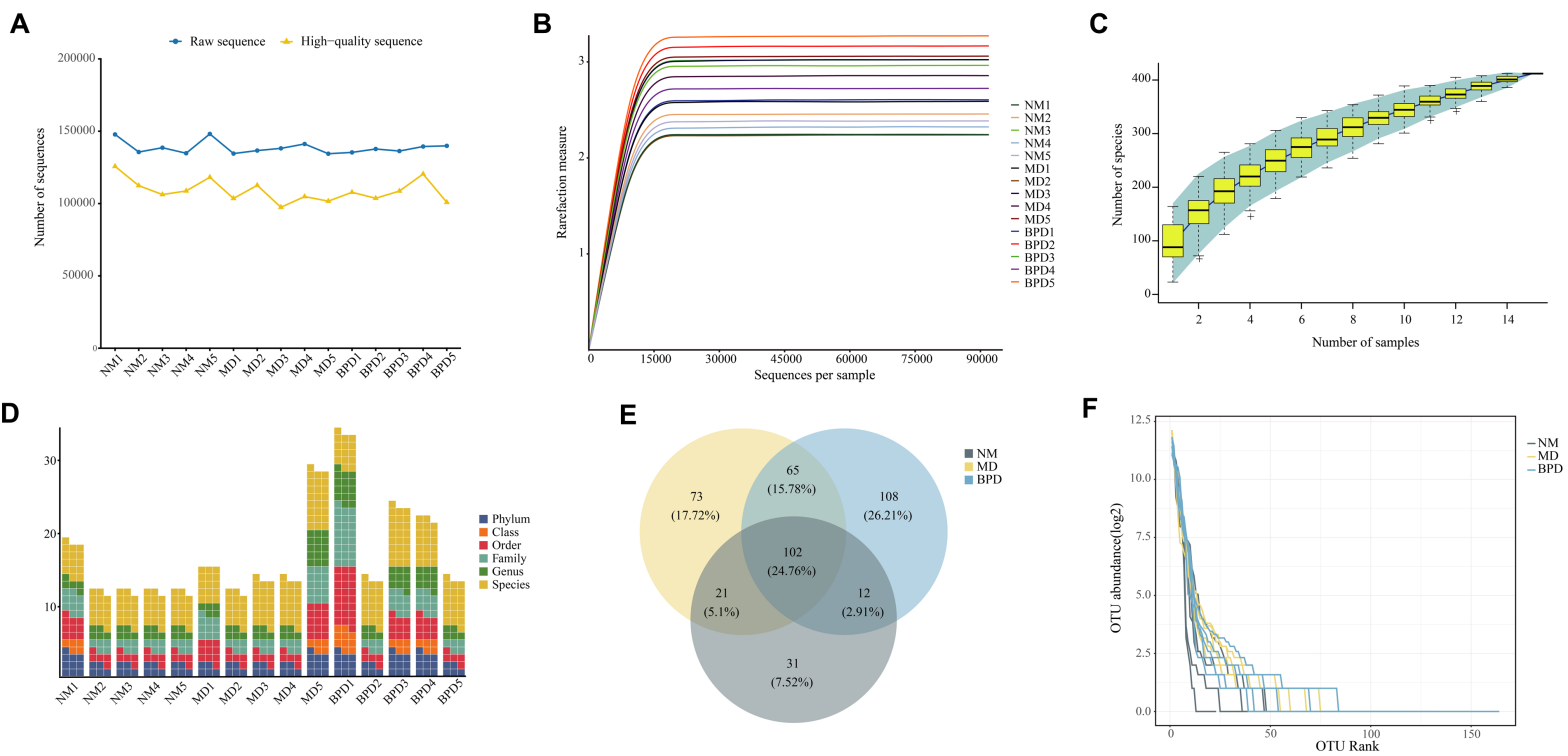
Intestinal mucosa sequencing quality assessment and number of OTUs. **(A)** Number of sequences. **(B)** Dilution curve of Shannon. **(C)** Species accumulation curve. **(D)** Number of OTUs at each taxonomic level. **(E)** Venn diagram. **(F)** Rank abundance distribution curve. The steeper the fold line in the horizontal axis, the lower the evenness of the community. NM, normal group; MD, model group; BPD, Baohe pill decoction group.

After sequencing was completed, 2,078,695 raw sequences were obtained (Figure 3A), and 1,632,715 high-quality sequences were obtained after screening for subsequent analysis. The rarefaction curve of Shannon shows that with increasing sequencing depth, the curve growth saturates when the sequencing depth reaches 15,000 (Figure 3B). Similarly, the number of observed species flattened out with increasing sample size, indicating that the sequencing depth and sample size in this study were the sufficient and further increase in sequencing depth had little gain for the next analysis (Figure 3C). In addition, there was some variability in the number of OTUs obtained for different taxonomic classes (Figure 3D). In Figure 3E, the number of OTUs delineated for each of the NM, MD, and BPD groups was 166, 261, and 287, respectively, and the common number of OTUs for the three groups was 102. We plotted the abundance rank curve using the log2 of OTU abundance as the vertical coordinate and the OTU abundance sorted from largest to smallest as the horizontal coordinate, and the length of the curve at the horizontal coordinate reflected the number of OTUs for that sample at that abundance. In the Figure 3F, the BPD group fold line spans the largest horizontal axis, and combined with the size of the number of OTUs possessed by each group, it tentatively indicates that both HFHPD and BPD interventions increased the richness of lactase-producing bacterial.

### 3.4. Effect of BPD on alpha diversity of lactase-producing bacteria in the intestinal mucosa of mice with diarrhea induced by HFHPD

**Figure 4:**
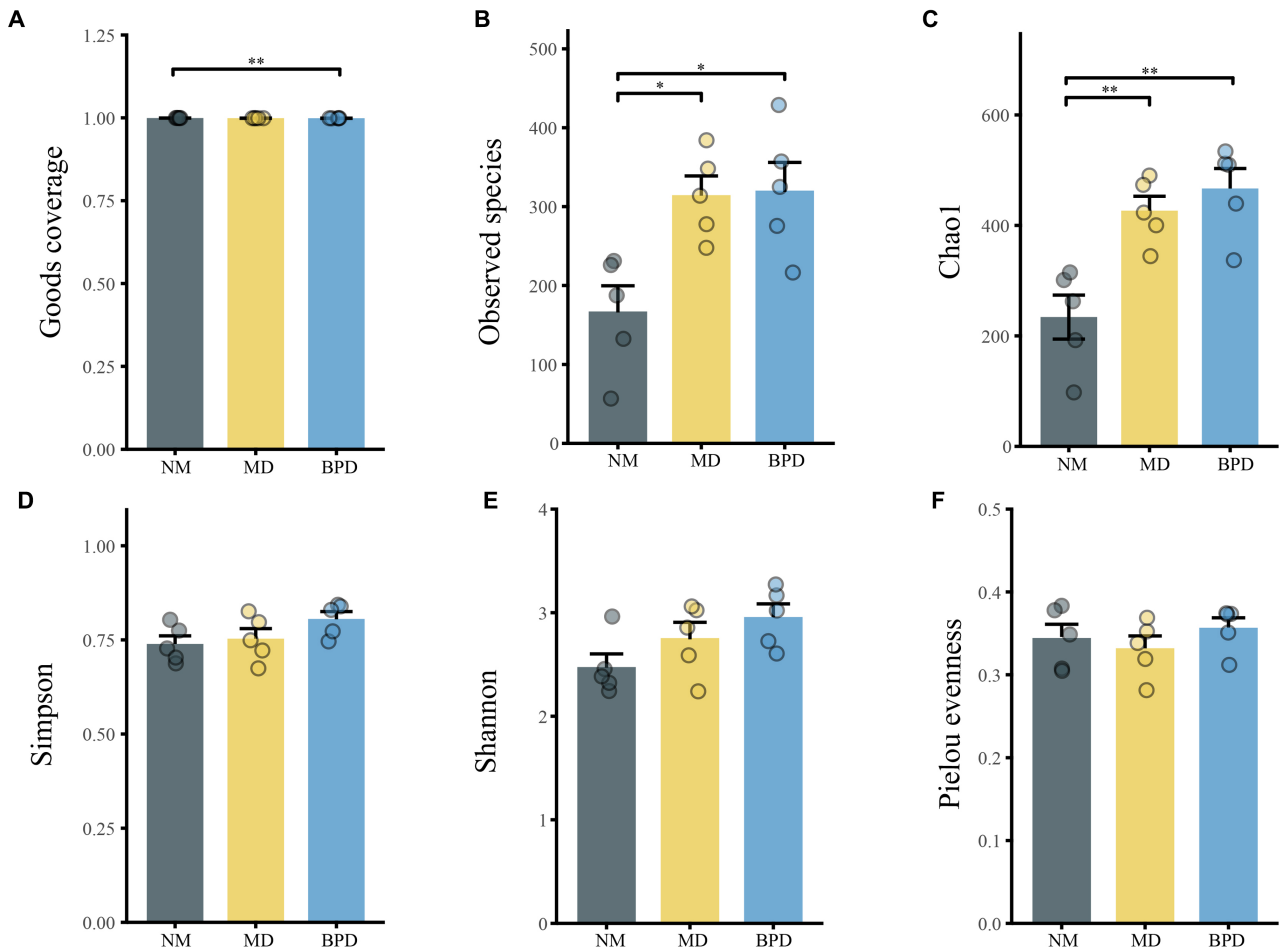
Alpha diversity of lactase-producing bacteria of intestinal mucosa in mice. **(A)** Goods coverage. **(B)** Observed species. **(C)** Chao1. **(D)** Simpson. **(E)** Shannon. **(F)** Pielou evenness. NM, normal group; MD, model group; BPD, Baohe pill decoction group. ^*^p < 0.05, ^**^p < 0.01.

In Figure 4, the Goods coverage index was close to 1 in all groups, indicating that the number of detected species had adequately covered the current sample. Compared with the NM group, the MD group mice had higher Chao1 (*p* < 0.01), Observed species (*p* < 0.05), Simpson (*p* > 0.05), Shannon (*p* > 0.05) indices, and lower Pielou evenness index (*p* > 0.05). After the intervention of Bohol Pill soup, the Observed species, Simpson, Shannon, and Pielou evenness indices of mice in the BPD group showed an increasing trend, but none of them was significant (*p* > 0.05). This indicates that the richness of lactase-producing bacteria in the intestinal mucosa of mice significantly increased after HFHPD modeling, while BPD had a slight promotion effect on the richness and diversity of lactase-producing bacteria in the intestinal mucosa of mice with diarrhea induced by HFHPD.

### 3.5. Effect of BPD on beta diversity of lactase-producing bacteria in the intestinal mucosa of mice with diarrhea induced by HFHPD

**Figure 5:**
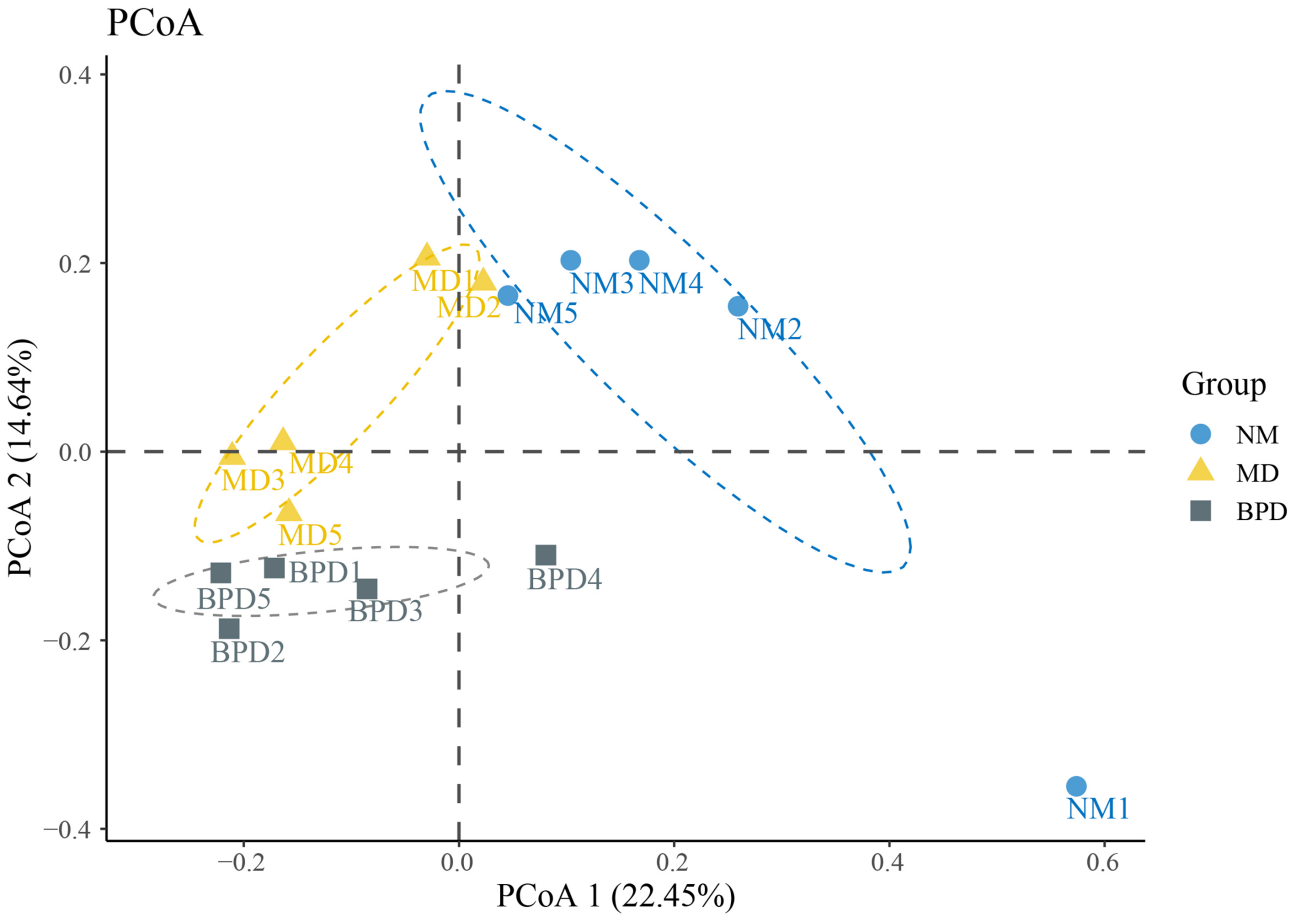
PCoA analysis. The larger the distance between the points in the graph, the larger the difference in species composition. NM, normal group; MD, model group; BPD, Baohe pill decoction group.

With PCoA, we can naturally decompose and rank the community data, which, in turn, allows us to assess the differences in the distribution of community structure. In PCoA, the closer the samples are, the more similar their sample structures are indicated. As shown in Figure 5, the contributions of PCoA1 and PCoA2 were 22.45% and 14.64%, respectively. Except for NM1, which was separated farther, both NM and BPD groups could be well clustered together individually. Meanwhile, NM, MD and BPD groups were all significantly separated, and the difference was found to be highly significant by Adonis test (*p* < 0.01). The above results suggest that both HFHPD modeling and BPD interventions changed the community structure of lactase-producing bacteria in the intestinal mucosa.

### 3.6. Effect of BPD on the taxonomic composition of lactase-producing bacteria in the intestinal mucosa of mice with diarrhea induced by HFHPD

**Figure 6:**
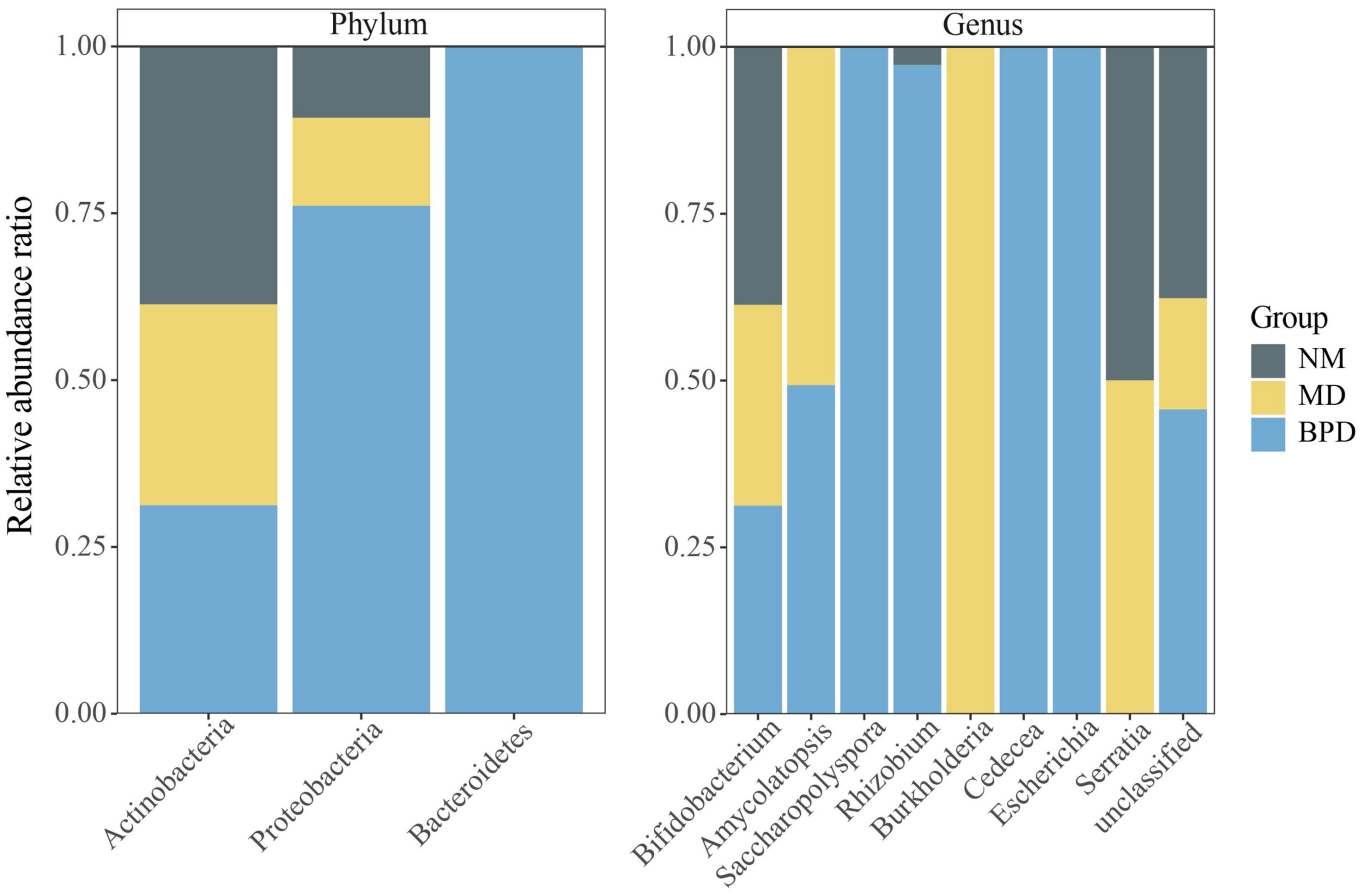
Percentage-filled column plot of relative abundance of lactase-producing bacteria in different groups. NM, normal group; MD, model group; BPD, Baohe pill decoction group.

The compositions of the three groups of lactase-producing bacteria are shown in Table 2. At the same time, we draw a percentage-filled column chart based on the relative abundance of each phylum or genus in the three groups (Figure 6). The lactase-producing bacteria in the intestinal mucosa of mice are mainly derived from Actinobacteria and Proteobacteria. At the genus level, 2, 4, and 8 lactase-producing bacterial genera were annotated in the NM, MD, and BPD groups, respectively, consistent with the highest richness in mice in the BPD group in Alpha diversity. Among them, *Bifidobacterium* was the most abundant genus, and the abundance ranking among the groups was NM > MD > BPD. In Figure 6, compared with the NM group, the MD group added new sources of lactase-producing bacteria from *Amycolatopsis*, *Burkholderia*, and *Serratia*. After intervention with BPD, the abundance of *Bifidobacterium*, *Amycolatopsis*, *Burkholderia*, and *Serratia* decreased, while the abundance of *Saccharopolyspora*, *Rhizobium*, *Cedecea*, and *Escherichia* increased in the BPD group.

**Table 2:** Taxonomic composition at phylum and genus levels.

	NM	MD	BPD
Actinobacteria	0.999973 ± 0.000023	0.999958 ± 0.000074	0.999779 ± 0.000216
Proteobacteria	0.000027 ± 0.000023	0.000042 ± 0.000074	0.000214 ± 0.000208
Bacteroidetes	0	0	0.000007 ± 0.000009
*Bifidobacterium*	0.999921 ± 0.000104	0.999871 ± 0.000212	0.999651 ± 0.000337
*Amycolatopsis*	0	0.000071 ± 0.000143	0.000064 ± 0.000092
*Saccharopolyspora*	0	0	0.000004 ± 0.000189
*Rhizobium*	0.000003 ± 0.000007	0	0.000123 ± 0.000000
*Burkholderia*	0	0.000008 ± 0.000016	0
*Cedecea*	0	0	0.000045 ± 0.000089
*Escherichia*	0	0	0.000002 ± 0.000004
*Serratia*	0	0.000004 ± 0.000008	0
unclassified	0.000076 ± 0.000097	0.000045 ± 0.000060	0.000112 ± 0.000113

NM, normal group; MD, model group; BPD, Baohe pill decoction group.

### 3.7. Correlation analysis of microbial and lactase activity with lactase-producing bacteria in the intestinal mucosa

**Figure 7:**
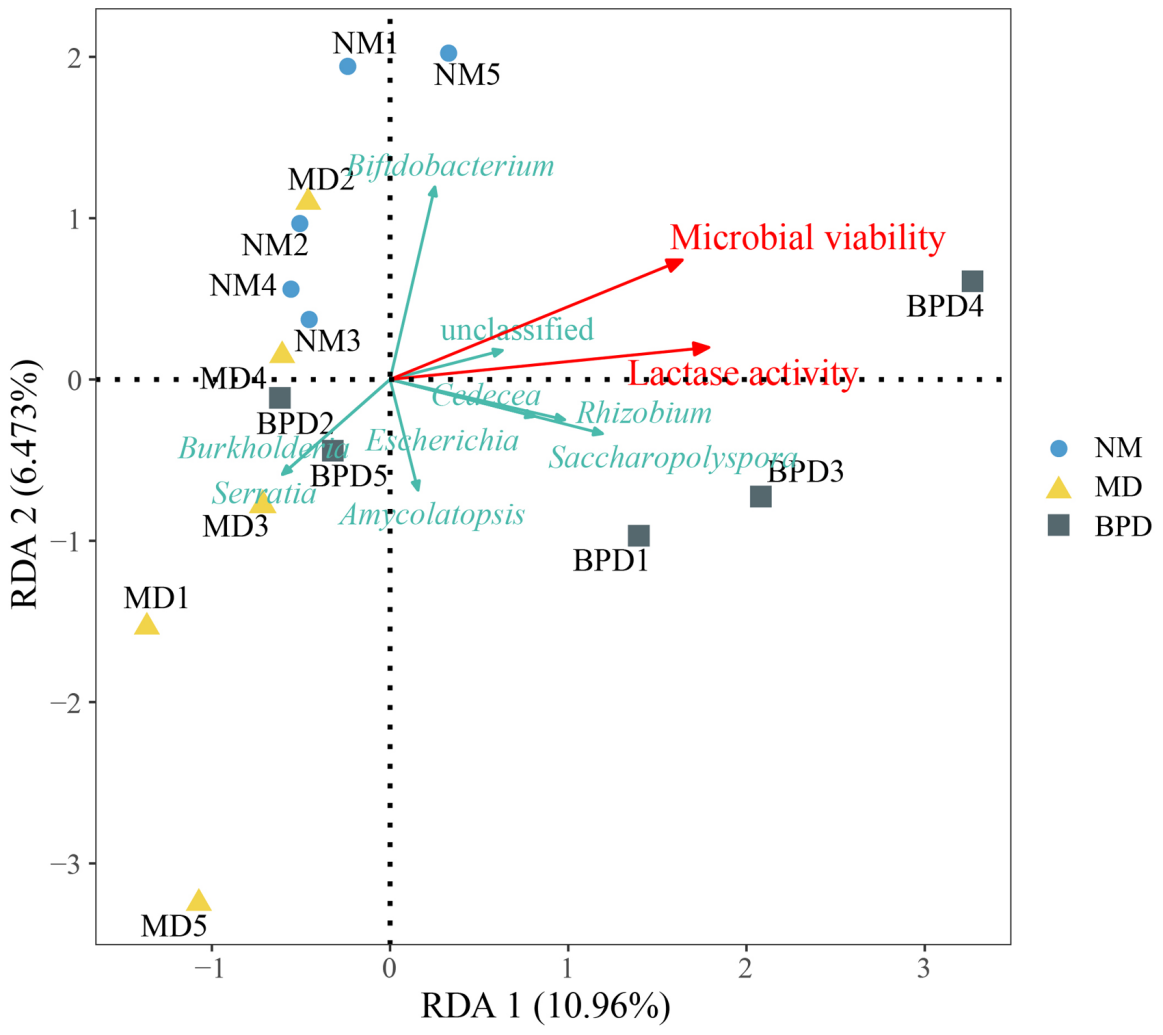
RDA analysis. The length of the arrow indicates the importance of this factor, and the angle between the arrows indicates the correlation between them. The acute angle between the arrows indicates a positive correlation, and the obtuse angle indicates a negative correlation. NM, normal group; MD, model group; BPD, Baohe pill decoction group.

We analyzed the relationship between microbial and lactase activity with lactase-producing bacteria in the intestinal mucosa by redundancy analysis (RDA). As can be seen from Figure 7, it can be seen that microbial and lactase activity was positively correlated with some bacteria, including *Bifidobacterium*, *Saccharopolyspora*, *Escherichia*, *Rhizobium,* and *Cedecea*. Microbial and lactase activity was negatively correlated with *Burkholderia* and *Serratia*.

## 4. Discussion

The intestinal microbiota is not only a deep participant in the digestive cycle and immune regulation, but also acts as a bridge between the human intestine and various physiological activities (Lin et al., 2023). The intestinal microbiota composition is profoundly influenced by dietary patterns, of which HFHPD may not be a diet model worth promoting (Farré et al., 2020). On the one hand, a high-fat diet reduces the expression of intestinal tight junction proteins, induces IEC oxidative stress and apoptosis, and decreases barrier-forming cytokines. This increases intestinal mucosal permeability, leading to intestinal barrier damage and endotoxemia expansion in the intestinal lumen (Rohr et al., 2020). On the other hand, moderate protein intake is highly beneficial, but excessive protein intake can be equally detrimental to health. For example, low protein levels and balanced amino acids can reduce nutritional diarrhea in piglets, but high-protein-induced amino acid imbalance can disrupt intestinal morphology and increase the risk of diarrhea (Han et al., 2016; Mittendorfer et al., 2020). Meanwhile, undigested proteins and amino acids can act as substrates for the growth of pathogenic bacteria, leading to increased diarrhea (Zhao et al., 2019).

The FDA method assesses microbial activity in a sample by detecting the activity of non-specific hydrolytic enzymes secreted by microorganisms, which naturally include lactase-producing microorganisms. Thus, the variation in microbial activity is somewhat representative of the activity of lactase-producing microorganisms. As part of the composition of the intestinal mucosal barrier, a decrease in the overall microbial activity inevitably affects the function of the intestinal mucosa. This is also evidenced by the trend in lactase activity in the intestinal mucosa that is consistent with microbial activity. In addition, the functional integrity of the intestinal mucosa is an important guarantee for the intestinal enzymes to play their role in the digestion and absorption of nutrients, and its impairment is detrimental to the expression of lactase activity in the organism (Wang et al., 2020). Compared with the NM group, intestinal mucosal microbial and lactase activity decreased significantly by HFHPD intervention and increased significantly by BPD intervention. Therefore, this means that the pathological mechanism of diarrhea due to HFHPD is associated with the downregulation effect of microbial and lactase activity of intestinal mucosa, while the therapeutic mechanism of BPD is closely related to the promotion of upregulation of intestinal microbial lactase activity.

The analysis of bacterial lactase genes allowed us to investigate further the effect of BPD on intestinal mucosal lactase-producing bacteria. Alpha diversity and OTU numbers showed that the diversity and abundance of intestinal mucosal lactase-producing bacteria increased after HFHPD intervention, which is consistent with our previous study on intestinal mucosal lactase-producing bacteria, but contrary to the change in diversity in intestinal contents. Due to the intestinal mucosal barrier and differences in the eco-regional environment, the intestinal mucosal microbiota may be more resistant and self-recover than the intestinal content microbiota (Zhou et al., 2022a). For example, Long et al. (2018b) reported a decrease in the abundance of lactase-producing bacteria in the intestinal contents of AAD mice, while the diversity and abundance of lactase-producing bacteria in the intestinal mucosa increased instead. Therefore, HFHPD intervention may have caused adaptive changes or translocation of lactase-producing bacteria in the intestinal mucosa. However, the change in the diversity of lactase-producing bacteria may not be the main mechanism of BPD in treating diarrhea induced by HFHPD. In our results, the richness and diversity of lactase-producing bacteria in the intestinal mucosa showed a small increase after the BPD intervention. Notably, the structure of lactase-producing bacteria was significantly altered. At the same time, the structure of intestinal mucosal lactase-producing bacteria in the BPD group was also significantly different from that of lactase-producing bacteria in the NM group of mice. This may be related to the rebound of intestinal mucosal lactase activity after BPD intervention. It is well known that lactase activity varies among bacterial sources. Compared to the MD group, *Saccharopolyspora*, *Rhizobium*, *Cedecea*, and *Escherichia*, all of which have increased abundance in the BPD group, have varying degrees of lactase production capacity. However, their contribution to host lactase activity is not outstanding (Harada et al., 1994; Leahy et al., 2001; Liu et al., 2020). Compared to these lactase-producing bacteria, *Bifidobacterium* is recognized as a safe and reliable source of microbial lactase. Some lactases can also bind galactose residues to lactose receptors to synthesize galactooligosaccharides2 that can have the ability to stimulate the growth of probiotic bacteria such as *Bifidobacterium*, thus creating a virtuous cycle (Füreder et al., 2020; Azcarate-Peril et al., 2021; Mulualem et al., 2021). This is also evidenced by the positive correlation of *Bifidobacterium* with microbial activity and lactase activity in correlation analyses, but *Bifidobacterium* has a more complex association with diarrhea. Natural plant products or compound preparations have attracted extensive attention at home and abroad due to their multi-channel, multi-level, and multi-target action pathways (Khan et al., 2020; Li C. R. et al., 2022; Li X. Y. et al., 2022). In a previous study, BPD promoted *Bifidobacterium* abundance in intestinal contents and feces (Guo et al., 2022). But *Bifidobacterium* abundance in the intestinal mucosa did not increase after BPD intervention. The complex cross-feeding network provides stability to the microbiota metabolism of substances in the gut. If a bacterium with a specific enzyme gene is inhibited, another bacterium with a homologous enzyme gene can perform a top-down (Aakko et al., 2020; Meijer et al., 2020). However, the deletion or mutation of one of the contributing bacteria can still profoundly alter the way the host interacts with the ingested substance (Wardman et al., 2022). Therefore, the modulatory effect of Bohol Pill tonics on lactase activity may be related to the adjustment of the overall structure of lactase-producing bacteria in the intestine.

Finally, this study has some limitations. The amplification primers may need further improvement to avoid some important intestinal lactase-producing bacteria not being detected, such as *Lactobacillus* spp., which is widely colonization in the intestine (Behera et al., 2018). In addition, the source of lactase is rich, and except for bacteria, some fungi are also lactase-producing. Further additional validation work on lactase-producing fungi in the intestine is necessary for the future.

## 5. Conclusion

HFHPD reduced intestinal mucosal microbial and lactase activity in mice, while BPD probably improved lactase activity by altering the structure of lactase-producing bacteria in the intestinal mucosal microbiota, alleviating diarrhea.

## Data availability statement

The datasets presented in this study can be found in online repositories. The names of the repository/repositories and accession number(s) can be found at: https://www.ncbi.nlm.nih.gov/, PRJNA878615.

## Ethics statement

The animal study was reviewed and approved by the Animal Experiment Center of Hunan University of Chinese Medicine.

## Author contributions

KZ: writing manuscripts and data analysis. NX and MP: experimental design, funding acquisition, and supervision. KZ and XY: data collection. ZT: critical review. All authors contributed to the article and approved the submitted version.

## Funding

This work was supported by the Natural Science Foundation of Hunan Province (no. 2020JJ4468) and Postgraduate Innovation Project of Hunan University of Chinese Medicine (2022CX92).

## Conflict of interest

The authors declare that the research was conducted in the absence of any commercial or financial relationships that could be construed as a potential conflict of interest.

## Publisher’s note

All claims expressed in this article are solely those of the authors and do not necessarily represent those of their affiliated organizations, or those of the publisher, the editors and the reviewers. Any product that may be evaluated in this article, or claim that may be made by its manufacturer, is not guaranteed or endorsed by the publisher.
